# Consumer-facing Diagnostic Sensors in a Patient with Implantable Cardioverter-Defibrillator

**DOI:** 10.19102/icrm.2019.100907

**Published:** 2019-09-15

**Authors:** Anjali M. Doshi, Rebecca M. Ebert, James D. Grinnell, Leslie A. Saxon

**Affiliations:** ^1^Center for Body Computing, Keck School of Medicine, University of Southern California, Los Angeles, CA, USA; ^2^Division of Cardiovascular Medicine, Keck School of Medicine, University of Southern California, Los Angeles, CA, USA

**Keywords:** Apple Watch, atrial fibrillation, consumer-facing, implantable cardioverter-defibrillator, smartwatch

## Abstract

Since its introduction in 2015, the Apple Watch (Apple Inc., Cupertino, CA, USA) has been purchased by an estimated 60 million consumers and boasts algorithms cleared by the United States Food and Drug Administration able to detect bradycardia, tachycardia, and atrial fibrillation, with the newest version of the device also allowing for real-time electrocardiogram acquisition. This case offers potentially the first demonstration of an Apple Watch correctly detecting atrial fibrillation with an implantable cardioverter-defibrillator confirming the accuracy of the detection from stored electrograms.

## Introduction

Physicians and consumers alike have had access to United States Food and Drug Administration (FDA)–approved, on-demand smartphone-enabled electrocardiogram (ECG) acquisition and arrhythmia interpretation for several years.^1^ The use cases and accuracy of these devices have been well-documented, but further validation and the creation of patient-driven care paradigms are still needed.^2^ The concurrent widespread adoption of consumer activity sensors, now estimated to have been purchased by 20% of Americans, has primed the consumer to continuously assess their health status.^3^ Since its introduction in 2015, the Apple Watch (Apple Inc., Cupertino, CA, USA) has been purchased by an estimated 60 million consumers and has both unregulated and FDA-cleared algorithms that use photoplethysmograph signals to detect bradycardia (< 40 bpm), tachycardia (> 120 bpm), and atrial fibrillation (AF).^4^ Additionally, the newest incarnation of the device, the Apple Watch Series 4, allows for real-time ECG acquisition, and Apple is expected to sell about 24 million of these watches in 2019 alone.^5^

Nominally, the Apple Watch detects heart rate variability by creating a tachogram—a plot of the time between heartbeats—every two to four hours.^5^ For Series 1 through 4 watches, an irregular rhythm notification algorithm enables arrhythmia detection.^5^ However, the Series 4 watches also boast a real-time ECG capability that uses two dry electrodes on the back of the watch and crown to classify the wearer’s heart rhythm on a 30-second ECG with PDF portability. The recently reported results of the Apple Heart Study studied the accuracy of the algorithm (the study did not leverage the ECG) and explored how consumer-notified detection of AF can be managed in a virtual care flow scheme.^6^ During an eight-month period, the study enrolled 419,297 participants (Apple Watch Series 0–3). A total of 2,161 (0.5%) participants had an irregular pulse notification suggestive of AF (mean age: 41 ± 13 years). The rate in patients aged 65 years or older was 3.2%. In subjects who received a notification and wore an ECG patch monitor for a mean wear time of 6.3 days, 34% had identified AF. The study found that, in patients with simultaneous watch and patch monitoring, the algorithm correctly identified AF 84% of the time (positive predictive value). There were no application-related adverse events, indicating the use of the Apple Watch for the diagnosis of AF to be a safe option for all patients.

## Case presentation

A 57-year-old Filipino male was diagnosed with idiopathic cardiomyopathy in 2002 and experienced reverse remodeling and marked symptom improvement on guidelines-indicated heart failure medications. He worked full time as a dentist and was able to play tennis without limitations until 2011, when he experienced a witnessed cardiac arrest at home. He received cardiopulmonary resuscitation (CPR) from his wife and the observed ventricular fibrillation was defibrillated by paramedics, and he subsequently made a full recovery. Shortly after his admission, he underwent dual-chamber implantable cardioverter-defibrillator (ICD) placement. Subsequent genetic testing for an inherited arrhythmia syndrome was negative, and he returned to work and became an avid golfer.

**Figure 1:**
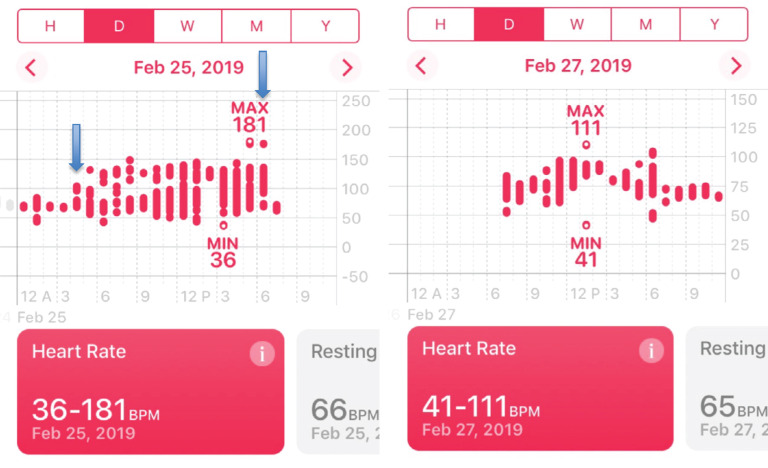
**A:** Apple heart rate histogram demonstrating variability of the heart rate consistent with an irregular response in AF (blue arrow). **B:** Apple heart histogram demonstrating less detection of heart rate variability, consistent with a normal or paced rhythm.

**Figure 2:**
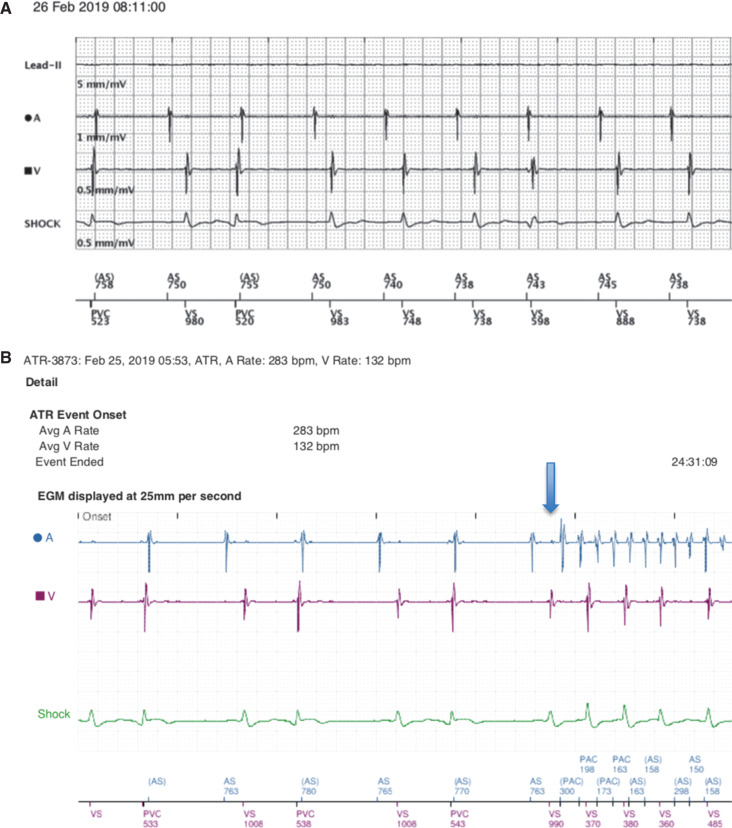
**A:** Stored ICD electrogram demonstrating AF that coincided with the February 25, 2019 Apple Watch notification (blue arrow). **B:** ICD electrogram recorded in the clinic on February 26, 2019, demonstrating a return to normal sinus rhythm.

On February 25, 2019, while playing golf and wearing his Apple Watch Series 3, the watch notified him through a haptic alert that his heart rhythm was irregular. He was asymptomatic, but, later that day, he felt some minimal increase in his heart rate. The following morning, he felt well and used the Apple Health app to determine that his heart rates were more regular than when he received the AF alert **(Figure 1)**. At a routine clinic appointment the day after the event, ICD interrogation revealed that the patient did experience an AF episode that spontaneously converted **(Figure 2)**. His Apple Watch had thus correctly detected the AF, and the evaluation of heart rate data from the Health app clearly showed a difference in heart rate regularity.

## Discussion

To our knowledge, this is the first demonstration of an Apple Watch correctly detecting AF in a patient with an ICD, confirming the accuracy of the detection from stored electrograms.

The use of a cardiac rhythm management device to validate wearable ambulatory monitors has been demonstrated previously.^7^ Wasserlauf et al. compared the accuracy of AF detection of an Apple Watch paired with a KardiaBand (AliveCor Inc., San Francisco, CA, USA) in comparison with an implantable cardiac monitor. When applied to episodes lasting more than one hour, the smartwatch detected 80 out of 82 episodes, achieving a sensitivity rate of 97.5%. However, neither device has the capability of including an atrial pacing lead to record intracardiac signals, an update that may improve the accuracy of validation.

In a separate study, Hwang et al. confirmed the accuracy of the heart rate detection of induced supraventricular tachycardia in the electrophysiology laboratory^8^ during a comparison of the Apple Watch Series 2; Samsung Galaxy Gear 3 (Samsung, Seoul, South Korea); and Fitbit Charge 2 (Fitbit, Inc., San Francisco, CA, USA). In 51 consecutive patients, each randomly assigned to wear two of the watches, each device was proven to be highly accurate in the detection of supraventricular tachycardia. However, this extremely accurate detection of fast regular rhythms in the presence of invasive catheters does not account for some of the challenges inherent in the detection of irregular rhythms such as AF to be addressed.

## Future directions

The present case illustrates how patient-facing diagnostic tools can drive earlier treatment and potentially improve outcomes in symptomatic and asymptomatic patients alike. Validated algorithms such as those that present in the Apple Watch can serve as patient-facing screening tools to facilitate appropriate treatments. Further, consumer-facing medical-grade screening tools help to enhance patient awareness and engagement regarding their condition. In the future, patient-facing on-demand educational tools should also be made available so that patients themselves can drive high-quality care, such as allowing access to novel oral anticoagulants when appropriate. Leveraging the patient in their own care, especially with accompanying educational content, presents the potential to ensure enriched and improved patient–physician engagement when an in-person visit does occur. For example, the University of Southern California’s Virtual Care Clinic studies how digital care, enabled by implantable, body-worn, or smartphone-based diagnostic sensors and software applications, promotes greater patient education and engagement in care. Our belief is that this model permits physicians to better practice “at the top of their license” because leveraging the patient in their own diagnosis and care is efficient, frees the physician from needing to perform time-constraining nominal tasks, and allows more time for personalized care plans. This clinical care model has great potential to enhance patients’ and physicians’ experiences.^9^ More research needs to be done to define this model and confirm that it results in improved outcomes.
